# The Relationship between the Blood Pressure Responses to Exercise following Training and Detraining Periods

**DOI:** 10.1371/journal.pone.0105755

**Published:** 2014-09-10

**Authors:** Emily A. Moker, Lori A. Bateman, William E. Kraus, Linda S. Pescatello

**Affiliations:** 1 Department of Kinesiology, University of Connecticut, Storrs, CT, United States of America; 2 Division of Cardiology, Department of Medicine, Duke University Medical Center, Durham, NC, United States of America; University of British Columbia, Canada

## Abstract

**Background:**

Exercise training lowers blood pressure (BP), while BP increases and returns to pre-training values with detraining. Yet, there is considerable variability in these BP responses. We examined the relationship between the BP responses after 6 months of training followed by 2 weeks of detraining among the same people.

**Methodology/Principal Findings:**

Subjects (n = 75) (X+SD, 50.2±10.6 yr) were sedentary, obese, and had prehypertension. They completed an aerobic (n = 34); resistance (n = 28); or aerobic + resistance or concurrent (n = 13) exercise training program. We calculated a metabolic syndrome *z* score (MetSz). Subjects were classified as BP responders (BP decreased) or non-responders (BP increased) to training and detraining. Linear and multivariable regression tested the BP response. Chi Square tested the frequency of responders and non-responders. The systolic BP (SBP, r = −0.474) and diastolic (DBP, r = −0.540) response to training negatively correlated with detraining (p<0.01), independent of modality (p>0.05). Exercise responders reduced SBP 11.5±7.8 (n = 29) and DBP 9.8±6.2 mmHg (n = 31); non-responders increased SBP 7.9.±10.9 (n = 46) and DBP 4.9±7.1 mmHg (n = 44) (p<0.001). We found 65.5% of SBP training responders were SBP detraining non-responders; while 60.9% of SBP training non-responders were SBP detraining responders (p = 0.034). Similarly, 80.6% of DBP training responders were DBP detraining non-responders; while 59.1% of DBP training non-responders were DBP detraining responders (p<0.001). The SBP detraining response (r = −0.521), resting SBP (r = −0.444), and MetSz (r = 0.288) explained 44.8% of the SBP training response (p<0.001). The DBP detraining response (r = −0.553), resting DBP (r = −0.450), and MetSz (r = 0.463) explained 60.1% of the DBP training response (p<0.001).

**Conclusions/Significance:**

As expected most subjects that decreased BP after exercise training, increased BP after detraining. An unanticipated finding was most subjects that increased BP after exercise training, decreased BP after detraining. Reasons why the negative effects of exercise training on BP maybe reversed with detraining among some people should be explored further.

**Trial Registration Information:**

ClinicalTrials.gov 1R01HL57354; 2003–2008; NCT00275145

## Introduction

Hypertension is one of the most important cardiovascular disease (CVD) risk factors [1], [2], and is the most common primary diagnosis in the United States [3]. Once blood pressure (BP) becomes higher than normal, there is a rapid progression to established hypertension. For, one in five people with prehypertension develop hypertension within 4 years [4], [5]; and most American will acquire hypertension if they live into old age [1].

Aerobic exercise training lowers resting BP 5 to 7 mmHg, while resistance exercise training lowers BP 2 to 3 mmHg among people with hypertension [6]. The BP lowering effects of exercise training occur rapidly within just three exercise sessions, persist with continued training, and diminish to pre-training levels within 2 weeks after training has ceased [7], [8]. For these reasons, the American College of Sports Medicine recommends individuals with hypertension engage in moderate intensity, aerobic exercise on most days of the week for 30–60 minutes per day, and moderate intensity, resistance training two to three days per week as a supplement to aerobic exercise training to lower BP [6], [9].

Despite the general consensus that exercise training lowers BP, there is considerable individual variability in the BP response to exercise training. We [10] and others [11], [12] have shown 20–25% of the people with hypertension do not lower BP following exercise. Furthermore, a percentage of these may adversely respond to exercise training as antihypertensive therapy, increasing BP 10 or more mmHg for reasons that are not clear [13]. We have completed several studies showing that resting BP and the components of the metabolic syndrome (MetS) as defined by the Adult Treatment Panel III [1] account for a clinically meaningful amount of the variability in the BP response to aerobic exercise [14]–[18]. In another study we showed that these effects may be dependent upon exercise modality [19]. Furthermore, the relationship between the BP response to exercise training and detraining has yet to be examined among the same people.

Therefore, the purpose of this study was to examine the BP responses after 6 months of exercise training followed by a 2 week detraining period among sedentary, middle-aged adults with prehypertension and mild to moderate dyslipidemia from the clinical trial, Studies of a Targeted Risk Reduction through Defined Exercise (STRRIDE-AT/RT) (1R01HL57354; 2003–2008; NCT00275145) [20]. We hypothesized the majority of STRRIDE-AT/RT participants would decrease their BP after 6 months of exercise training and increase their BP to pre-training levels after 2 weeks of detraining. However, there would be considerable variability in the BP responses to training and detraining that would be partially accounted for by resting BP and the MetS.

## Methods

This is a sub-study of the Studies of a Targeted Risk Reduction Intervention through Defined Exercise or STRRIDE-AT/RT (1R01HL57354; 2003-2008; NCT00275145)[19]–[22]. The two primary purposes of STRRIDE-AT/RT were to: 1) investigate the effects of different types and intensities of exercise training regimens on established CVD risk factors; and 2) to study the peripheral biologic mechanisms through which exercise training altered these CVD risk factors. Subjects were recruited from September 1, 2004 to December 31, 2008. We only describe the STRRIDE-AT/RT methods used in this sub-study below. However, the informed consent for this trial are available as Protocol Informed Consent S1.

### Subjects

Volunteers were men and women 18–70 years who were exercising ≤2 d·wk^−1^ or had a peak oxygen uptake (VO_2_peak) ≤35 mL·kg^−1^·min^−1^. Study inclusion criteria were: (a) body mass index (BMI) 25–35 kg·m^−2^; (b) resting systolic BP (SBP) <160 mmHg and/or diastolic BP (DBP) <90 mmHg; and (c) fasting low density lipoprotein-cholesterol (LDL) 130–190 mg·dL^−1^; or fasting high density lipoprotein-cholesterol (HDL) ≤40 mg·dL^−1^ for men and ≤45 mg·dL^−1^ for women. Subjects had no known metabolic, muscular, or coronary heart disease(s) and were not taking any antilipidemic or antihypertensive medications. Prior to participating in the study, individuals were screened by telephone and signed a written informed consent approved by the IRB of the participating institutions, Duke University Medical Center and East Carolina University. This sub-study did not meant the definition of " human subjects research" under Study Design Overview 45.CFR46.102(d) or 45.CFR. 46.102(f) at the University of Connecticut. Therefore, we were not required to file an application for review by the University of Connecticut.

Subjects attended a baseline assessment prior to the start of the 6 month exercise training intervention in which they completed a health screening questionnaire and a series of health fitness and anthropometric measurements. Pre-training measurements were performed at the completion of a 4 month wait list control period during which subjects were asked to maintain their current lifestyle. Subjects were then randomized into one of three exercise training programs: aerobic (AT) (n = 34), resistance (RT) (n = 28), or aerobic exercise and resistance concurrently (AT+RT) (n = 13). Measurements were performed again following the 6 month exercise training intervention. To be included in this sub-study, STRRIDE-AT/RT subjects had to have obtained BP measurements before (i.e, at the completion of the wait list control period) and after exercise training and the 2 week detraining period.

### Exercise Training Intervention

#### Aerobic

The AT group performed a peak cardiopulmonary graded exercise test before exercise training to determine the AT intensity. After a ramp period of 1 month, subjects exercised 3 d·wk^−1^ at 65–80% VO_2_peak on average 130±22.7 min·wk^−1^ for 5 months expending the caloric equivalent to walking or jogging 20 mi·wk^−1^. To ensure attainment of the proper AT intensity, subjects wore a heart rate monitor (Polar Electro, Inc., Woodbury, NY) at each session that was uploaded weekly. Subjects were instructed to use the treadmill as the primary mode to train, however, the bicycle ergometer, elliptical, and stair climber were other options.

#### Resistance

The RT group began with one set of 8 to 12 repetitions per set of eight exercises (four upper and four lower) targeting major groups for the first 2 weeks followed by two sets during weeks three and four until they reached the intended three sets during week five. Subjects exercised at 70–85% of their one repetition maximum 3 d·wk^−1^ for 5 months using Cybex weight machines (Cybex International Inc; Medway, MA). The Duke University Medical Center RT group used only Cybex machines, while the East Carolina University RT group used Cybex weight machines to perform the upper and lower body exercises, abdominal crunch exercises were also incorporated, and at week 14 subjects transitioned to free weights for the upper body exercises. Subjects who were able to perform three sets of 12 repetitions in two consecutive sessions increased resistance in 5 lb increments.

#### Aerobic and Resistance

The AT+RT group performed the identical training program as the AT and RT groups in combination.

#### Exercise Supervision

All training sessions were closely monitored. East Carolina University provided direct supervision, while Duke University Medical Center had direct supervision and/or used the FitLinxx Strength Training Partner (FitLinxx; Norwalk, CT). FitLinxx Strength Training Partneris a computer system designed to electronically track workouts and automatically send training results to the FitLinxx server.

#### Anthropometric Assessments

Height was measured to the nearest quarter cm and weight to the nearest 0.1 kg on a digital scale (Scale 5005; Scale Tronix Inc, Wheaton, IL) to calculate body mass index (BMI, kg·m^−2^). Waist circumference (WC) was taken around the abdominal waist at the iliac crest. Each measurement was taken twice to the nearest 0.1 cm and averaged.

#### Blood Pressure

A trained research nurse measured BP by auscultation on the non-dominant arm before and after training and detraining. Subjects sat quietly in the laboratory for 60 minutes with feet flat on the floor in the relaxed position. Two BP measurements were obtained 20–30 minutes apart and then averaged by a nurse trained in standard procedures. [23] Mean arterial pressure (MAP) was calculated as: DBP+0.33* (SBP-DBP).

#### Fasting Blood Sample Determinations

Subjects fasted for 12 hours. Total cholesterol, LDL, HDL, triglycerides (TG), glucose, and insulin were analyzed from fasting plasma using the nuclear magnetic resonance spectroscopy technique (LipoScience; Raliegh, NC).

#### Metabolic Syndrome Classification

Presence of the metabolic syndrome (MetS) was defined as having three or more of the following: triglycerides (TG) ≥150 mg·dL^1^, BP ≥130/85 mmHg, glucose ≥100 mg·dL^−1^, WC ≥102 cm for men and ≥88 cm for women, and/or HDL <40 mg·dL^−1^ for men and <50 mg·dL^−1^ for women [24].

The MetS z-score was a continuous standardized calculation of the five MetS components: HDL, TG, glucose, WC, and MAP [19]. Individual subject data, current ATP III criteria [24], and standard deviations (SD) from the entire STRRIDE-AT/RT cohort at baseline were used to calculate the MetS z-score. The equations used to calculate the MetS z score were: {z Score = [(40-HDL)/6.2]+[(TG-150)/66.2]+[(glucose-100)/10.4]+[(WC-102)/9.3]+[(MAP-100)/8.7]} for men; and {z Score = [(50-HDL)/11.8]+[(TG-150)/66.2]+[(glucose-100)/10.4]+[(WC-88)/9.2]+[(MAP-100)/8.7]} for women [19].

#### Cardiopulmonary Graded Exercise Test

All subjects completed a cardiopulmonary graded exercise test on a treadmill with a 12-lead electrocardiograph and had expired gas measured using a TrueMax 2400 Metabolic Cart (ParvoMEdics; Sandy, UT). The test protocol included 2 minute stages with the workload increasing by one metabolic equivalent per stage. VO_2_peak was determined by averaging the two consecutive highest 15 second readings. A respiratory exchange ratio ≥1.10 was the criteria used to terminate the test.

### Statistical Analysis

Descriptive statistics (mean±SD) were calculated for all variables. Repeated measures analysis of covariance was used to test the change in BP, MetS z score, and other individual components of the MetS after exercise training (i.e., after versus before training) and after detraining (i.e., after 2 weeks of detraining versus after training) by exercise groups (AT, RT, AT+RT) with gender as a fixed factor. Pre-training BMI and the MetS z score, and the change in VO_2_peak after training were covariates. Bonferroni post-hoc tests were used to test for BP differences among groups. Chi-Square was used to test the frequencies of individuals who decreased (i.e., responders, BP change <0 mmHg) or increased (i.e., non-responders, BP change ≥0 mmHg) BP after exercise training and detraining. Simple linear and multivariable regressions were performed to test for correlates of the BP response to exercise training with a variance inflation factor <2 used to indicate covariance among the independent variables was low [10].

There is considerable inter- and intra-variability in the response of cardiometabolic risk factors to exercise [12], [13]. Therefore, we used chi-square to test the frequencies of subjects whose BP decreased (i.e., responders, BP change <0 mmHg) or increased (i.e., non-responders, BP change ≥0 mmHg) and MetS profile improved (i.e., number of MetS components decreased) or became worse (i.e., number of MetS components increased) by summing the response of the individual components of the MetS after versus before exercise training. The MetS profile components used for these determinations were MAP, WC, HDL, triglycerides, and glucose to be consistent with the MetS z score equations [19]. All statistical analyses were performed with the Statistical Package for Social Science (SPSS) version 19.0 for Macintosh (IBM, Armok, NY) with p<0.05 established as the level of statistical significance.

## Results

### Subjects

The sub-study population consisted of 75 sedentary, middle aged, obese men (n = 38) and women (n = 37) with prehypertension, mild to moderate dyslipidemia, and normal fasting glucose (Table 1). In addition, 29.3% of the total sample had the MetS [24]; while 34.5% of SBP training responders, 26.1% of SBP training non-responders, 35.5% of DBP training responders, and 25.0% of DBP training non-responders had the MetS with no difference in prevalence between BP responders and non-responders (p>0.05). SBP training responders had a higher BMI (p = 0.025) and resting BP (p = 0.003) and lower glucose levels (p = 0.031) and VO_2_peak (p = 0.051) than SBP non-responders. DBP training responders had a higher resting BP compared to DBP non-responders (p = 0.025). All other characteristics were not different between SBP and DBP training responders and non-responders (p>0.05).

**Table 1:** Baseline subject characteristics (Mean±SD) of the total sample and by blood pressure exercise training responders and non-responders.

Variable	Total Sample (n = 75)	SBP		DBP	
		Responder (n = 29)	Non- responder (n = 46)	Responder (n = 31)	Non- responder (n = 44)
Age (yr)	50.2±10.6	52.6±12.0	48.7±9.5	50.2±11.0	50.3±10.4
SBP (mmHg)	120.0±13.7	125.8±14.3^†^	116.3±12.0	121.7±15.9	118.7±11.9
DBP (mmHg)	79.1±9.2	80.7±9.7	78.1±8.8	81.9±9.7*	77.1±8.4
BMI (kg/m^−2^)	30.5±3.2	31.5±2.7*	29.8±3.3	30.3±2.9	30.6±3.3
WC (cm)	97.0±9.8	97.3±9.8	96.7±9.9	97.5±10.0	96.5±9.7
CHOL (mg·dL^1^)	226.2±31.6	228.7±36.8	224.6±28.2	228.6±31.4	224.5±31.9
HDL (mg·dL^−1^)	52.1±15.0	55.2±14.6	50.2±15.1	51.8±16.6	52.4±13.9
LDL (mg·dL^−1^)	144.2±25.1	143.4±30.5	144.8±21.3	143.9±26.	144.4±24.6
TG (mg·dL^−1^)	153.3±85.7	150.0±68.6	155.4±95.5	171.8±101.2	140.3±71.2
Glucose (mg·dL^−1^)	92.4±11.5	88.8±13.9*	94.7±9.1	89.9±14.3	94.3±8.8
Insulin (mg·dL^1^)	8.9±4.5	9.2±5.7	8.7±3.6	8.5±4.6	9.3±4.5
VO_2peak_ (mL·kg^−1^·min^−1^)	28.1±6.5	26.3±5.6*	29.3±6.8	29.8±6.9	27.0±6.0

SBP, systolic blood pressure; DBP, diastolic blood pressure; BMI, body mass index; WC, waist circumference; CHOL, total cholesterol; HDL, high density lipoprotein; LDL, low density lipoprotein; VO_2peak_, peak oxygen uptake; MetS z score; metabolic syndrome z score.

*p<0.05, ^†^p = 0.001, Responder vs non-responder.

### The Blood Pressure Responses to Exercise Training and Detraining

#### Overall and by Exercise Modality

The change in SBP (0.4±10.6 mmHg) and DBP (−0.8±7.0 mmHg) after training was not different among the total sample (p>0.05); and the change in SBP/DBP was also not different among exercise groups, i.e., AT (n = 34) 1.9±10.8/0.1±6.0 mmHg, RT (n = 28) 2.5±8.3/−0.8±5.5 mmHg, and AT+/RT (n = 13) −4.0±10.1/−2.4±9.1 mmHg, respectively (p>0.05). The change in SBP (0.3±11.7 mmHg) and DBP (0.9±8.7 mmHg) after detraining was not different among the total sample (p>0.05); and the change in SBP/DBP was also not different among exercise groups, i.e., AT −1.4±11.0/−2.3±8.2 mmHg, RT 0.6±10.2/1.4±7.8 mmHg, and AT+RT 3.1±11.3/3.9±8.3 mmHg, respectively (p>0.05).

#### Responders versus Non-responders

Training responders decreased SBP (n = 29) by −11.5±7.8 mmHg and DBP (n = 31) by −9.8±6.2 mmHg; whereas non-responders increased SBP (n = 46) by 7.9±10.9 mmHg and DBP (n = 44) 4.9±13.5 after training (p<0.001). Furthermore, the SBP and DBP response differed between training responders and non-responders (p<0.001). Detraining responders tended to decrease SBP (n = 46) by −3.1±12.9 mmHg (p = 0.109) and decreased DBP (n = 44) by −3.4±7.5 mmHg (p = 0.005); whereas detraining non-responders tended to increase (n = 29) SBP by 6.5±16.7 mmHg (p = 0.051) and increased DBP (n = 31) by 5.3±12.9 mmHg (p = 0.031) after detraining. In addition, the SBP and DBP response was different between detraining responders and non-responders (p<0.001).

We found of the 29 people who were classified as SBP training responders (SBP decreased), 34.5% were SBP detraining responders (SBP decreased) and 65.5% were SBP detraining non-responders (SBP increased). Of 46 people who were classified as SBP training non-responders (SBP increased), 60.9% were SBP detraining responders (SBP decreased) and 39.1% were SBP detraining non-responders (SBP increased) (p = 0.034). Of the 31 people who were classified as DBP training responders (DBP decreased), 19.4% were DBP detraining responders (DBP decreased) and 80.6% were DBP detraining non-responders (DBP increased). Of the 44 people that were classified as DBP training non-responders (DBP increased), 59.1% were DBP detraining responders (DBP decreased) and 40.9% were DBP detraining non-responders (DBP increased) (p<0.001). Therefore, the majority of subjects that decreased BP after training (responders), increased BP after detraining (nonresponders); while the majority of subjects that increased BP after training (nonresponders), decreased BP after detraining (responders). Last, 62.1% of SBP training responders were also DBP training responders; whereas 52.2% of SBP detraining responders were also DBP detraining responders.

#### Predictors of Blood Pressure to Exercise Training

Table 2 displays the correlates of the BP response after training. Factors accounting for 44.8% of the variance in SBP response to training were the SBP response to detraining, resting SBP, and the change in the MetS z score after training (p<0.001). Factors accounting for 60.1% of the variance in the DBP to training were the DBP response to detraining, resting DBP, and the change in the MetS z score after training (p<0.001).

**Table 2:** Correlates of the blood pressure response after versus before 6 months of exercise training.

	Correlates	β	t	Partial r	r^2^	p
SBP	Detraining SBP	−.461	−5.149	−.521		<0.001
	Resting SBP	−.379	−4.171	−.444		<0.001
	MetS z score	.235	2.536	.288		0.013
Model Summary				.686	.448	<0.001
DBP	Detraining DBP	−.448	−5.595	−.553		<0.001
	Resting DBP	−.339	−4.245	−.450		<0.001
	MetS z score	.371	4.405	.463		<0.001
Model Summary				.786	.601	<0.001

SBP, systolic blood pressure; DBP, diastolic blood pressure; MetS z score; metabolic syndrome z score.

#### The Blood Pressure and Metabolic Syndrome Response to Exercise Training

The MetS z score was not different after exercise training among the total sample (p>0.05) (Table 3). The MetS z score decreased (i.e, improved) among SBP and DBP training responders after training (p<0.001), but was not different among SBP and DBP training non-responders (p>0.05). MAP was not different after exercise training among the total sample (p>0.05). MAP decreased among SBP and DBP training responders after training, and increased among SBP and DBP training non-responders (p<0.001). WC decreased after training among the total sample (p = 0.005) and SBP training non-responders (p = 0.010). HDL increased after training among DBP training non-responders (p = 0.005). TG decreased after training among the total sample (p = 0.003) and SBP training non-responders (p = 0.025).

**Table 3:** The response (Mean±SD) of the metabolic syndrome z score and the individual components of the metabolic syndrome z score after versus before 6 months of exercise training.

Component	Total Sample (n = 75)	SBP		DBP	
		Responder (n = 29)	Non-responder (n = 46)	Responder (n = 31)	Non-responder (n = 44)
Metabolic Syndrome z score	−0.5+2.0	−1.7+1.9^†&^	0.3+2.4	−1.4+2.9* ^&^	0.4+1.5
MAP (mmHg)	−0.7±9.5	−8.5±6.1^†&^	5.1±9.2^†^	−8.2±7.2^†&^	4.8±8.5^†^
WC (cm)	−1.2±3.6*	−1.1±3.4	−2.1±5.1*	−1.8±5.3	−0.6±3.6
HDL (mg·dL^−1^)	1.2±5.5	1.4±7.6	1.5±6.9	−1.9±7.9	2.1±4.6*
TG (mg·dL^−1^)	−20.4±58.2*	−25.7±66.0* ‡	−15.6±80.0	−31.5±72.9	−13.8±61.9
Glucose (mg·dL^−1^)	0.9±9.7	−0.1±11.2	1.7±13.7	−1.2±11.8	0.4±10.9

MAP, mean arterial pressure; WC, Waist Circumference; TG, Triglycerides.

*p<0.05, ^†^p<0.001 After versus before exercise.

‡ p<0.05, ^&^p<0.001, Responders versus non-responders.

We found of the 29 people classified as SBP training responders (SBP decreased), the MetS improved in 31.0% (number of components decreased) and the MetS became worse in 69.0% (number of components increased). Of the 46 people classified as SBP training non-responders (SBP increased), the MetS improved in 52.2% (number of components decreased) and the MetS became worse in 47.8% (number of components increased) (p = 0.059). Of the 31 people who were classified as DBP training responders (DBP decreased), the MetS improved in 29.0% (number of components decreased) and the MetS became worse in 71.0% (number of components increased). Of the 44 people classified as DBP training non-responders (DBP increased), the MetS improved in 54.5% (number of components decreased) and the MetS became worse in 45.5% (number of components increased) (p = 0.025). Thus, for the majority of subjects that decreased BP after training (responders), the number of components of the MetS increased or became worse after training.

## Discussion

The purpose of this STRRIDE-AT/RT sub-study was to examine the relationship between the BP response after 6 months of exercise training followed by a 2 week detraining period among 75 sedentary, middle aged men and women with prehypertension and mild to moderate dyslipidemia, 29.3% of which had the MetS. As hypothesized, the BP response after 6 months of exercise training was negatively correlated with the BP response following 2 weeks of detraining. Furthermore, the majority of SBP (65%) and DBP (80%) exercise training responders BP decreased) were SBP and DBP detraining non-responders (BP increased). An unanticipated finding was the majority of SBP (∼61%) and DBP (59%) exercise training non-responders (BP increased) were SBP and DBP detraining responders (BP decreased). The major correlates of the SBP and DBP response to exercise training were the BP response to detraining, resting BP, and the MetS z score, which accounted for 44.8% and 60.1% of the variability in the SBP and DBP response to exercise training, respectively. Last, the BP response to exercise training did not necessarily align with the response of the MetS to exercise training as the majority of subjects that decreased BP after training, experienced an increase in the number of MetS components after training.

The results of this sub-study provide new findings demonstrating that for a majority of middle aged men and women with prehypertension that experienced an adverse BP response to exercise training, these adverse BP effects were reversed following detraining. Furthermore, the BP response to exercise training may not align with the response of other cardiometabolic risk factors to exercise. Our findings are consistent with our reports [10], [14], [15], [17] and those of others [11], [12] indicating there is a clinically meaningful proportion of people that do not lower BP following exercise training. Recently, Bouchard et al. [13] consolidated the findings from six large exercise training studies that included STRRIDE-AT/RT to examine the variability in the response of cardiometabolic risk factors to exercise training. They found ∼12% of the participants experienced an adverse SBP response increasing SBP>10 mmHg, whereas a similar amount had an excellent response to exercise training decreasing SBP≤10 mmHg. Fasting insulin, TG, and HDL exhibited similar patterns of response, and similar to our findings, the response of these cardiometabolic risk factors was often different from the BP response to exercise training.

Insight into reasons for the unexpected finding of why some people experienced an adverse BP response to exercise training that was reversed with detraining may partially reside within the correlates of the BP response to exercise training that we found. The major predictors of the BP response to exercise training were the BP response to detraining, resting BP, and the MetS z score. These findings are consistent with our previous work showing resting BP and the MetS are important predictors of the BP response to exercise among sedentary, middle-aged, overweight men with pre- to Stage 1 hypertension [6], [10], [15]–[18]. Hypertension is a contributor to and component of the MetS [25]. Therefore, the mechanisms by which the MetS and its individual components would account for a clinically meaningful proportion of the BP response to exercise training may be partially explained by their apparent common underlying pathophysiology.

Erdogen et al. [26] examined the progression of prehypertension to established hypertension among 98 men and women 30 to 63 years with the MetS and prehypertension over 3 years. They found that the progression of prehypertension to hypertension positively correlated with resting SBP (p = 0.002) and the MetS (p = 0.009) among 98 men and women 30 to 63 years with the MetS and prehypertension over 3 years. Ferriera et al. [27] found apparently healthy individuals between 36 and 42 years with prehypertension at baseline and that had persistent MetS over 6 years exhibited the maladaptive arterial remodeling changes that were related to changes in SBP and DBP. However, these maladaptive arterial changes were reversed in individuals with prehypertension who recovered from the MetS. Collectively, these findings indicate that the MetS and its components should be explored further as clinical characteristics that may eventually be used to distinguish between individuals that respond and do not respond to exercise as antihypertensive therapy.

A limitation of this STRRIDE-AT/RT sub-study was its primary purpose was not the primary purpose of STRRIDE-AT/RT. Thus, our sub-study sample may have been underpowered to detect changes in BP as a result of exercise training as well as BP differences among the exercise training groups. Nonetheless, our sub-study sample was more than adequately powered to examine its primary purpose as the effect size to detect differences in the BP response after versus before 6 months of exercise training between responders and non-responders ranged from medium (0.341) to large (0.741). It is also possible that misclassification of BP responders and non-responders to exercise training and detraining may have occurred due to the inherent variability in BP measurement as well as regression to the mean [13], [28]. In order to minimize these biases, the research personnel collecting BP data were well trained clinicians in standard BP measurement protocols [23], each subject served as their own control, and multiple BP measurements were taken and averaged. Furthermore, the coefficient of variation among SBP and DBP before training and detraining approximated 5% indicating acceptable reproducibility.

In conclusion, the BP response after 6 months of exercise training was negatively correlated with the BP response following 2 weeks of detraining. Nonetheless, the majority of BP exercise training responders were BP detraining non-responders; whereas, the majority of BP exercise training non-responders were BP detraining responders. Furthermore, the BP response to exercise training did not necessarily align with the response of the other components of the MetS to exercise training. Further research is needed to elucidate reasons why detraining may reverse the adverse effects of exercise on BP for some individuals that may partially reside in the common underlying pathophysiology of hypertension and the MetS.

## Supporting Information

Protocol Informed Consent S1 From the Studies of a Targeted Risk Reduction through Defined Exercise (STRRIDE-AT/RT) (1R01HL57354; 2003–2008; NCT00275145).
(DOCX)
