# Supplementary material for: The Relationship between the Blood Pressure Responses to Exercise following Training and Detraining Periods
Source: PLoS One. 2014 Sep 10;9(9):e105755. doi: 10.1371/journal.pone.0105755 (PMC4160181; doi:10.1371/journal.pone.0105755)
Supplement: Protocol Informed Consent S1 — From the Studies of a Targeted Risk Reduction through Defined Exercise (STRRIDE-AT/RT) (1R01HL57354; 2003–2008; NCT00275145). (DOCX) [file pone.0105755.s001.docx]

You are being asked to take part in a research study in the Department of Medicine at Duke University Medical Center. As your study doctor or study staff discusses this consent form with you, please ask him/her to explain any words or information that you do not clearly understand. The nature of the study, inconveniences, discomforts, risks, and other important information are listed and described below.

**WHO WILL BE MY DOCTOR ON THE STUDY?**

Dr. William E. Kraus, M.D. will conduct the study. A grant from the National Institutes of Health will sponsor the study. Portions of Dr. Kraus’ and his research team’s salaries will be paid by this grant.

**WHY IS THE STUDY BEING DONE?**

Although regular exercise is known to improve exercise capacity and beneficially effect many factors that contribute to heart and blood vessel disease, significant controversy remains regarding the benefits of resistance training (e.g. weight lifting), either on its own or when combined with aerobic exercise. The purpose of the study is to evaluate the effects of different amounts and combinations of aerobic and resistance exercise training programs on exercise performance and risk factors for heart and blood vessel disease, including blood lipids (cholesterol and fatty acids), and blood glucose metabolism. An additional purpose of the study is to evaluate the factors in the blood and in exercising muscles that contribute to improvement in the risk factors. This information will be used to improve exercise training guidelines for improving cardiovascular health.

**HOW MANY PEOPLE WILL TAKE PART IN THE STUDY?**

Approximately one hundred and seventy-four (174) men and women volunteers will be recruited to participate in the study at Duke. An additional fifty-four (54) men and women will be recruited to participate at East Carolina University in Greenville, North Carolina.

In order to participate in the study the following inclusion criteria must be met:

- Age: 18-70 years
- Body Mass Index (BMI): 25.5 – 35.4
- Non-smoker
- Moderately elevated low density (LDL) cholesterol: >130 mg% – <190 mg% and/or moderately reduced high density (HDL) cholesterol: < 40 mg% in MEN, < 45 mg% in WOMEN
- Resting blood pressure: < 160/90 mmHg

The following criteria will exclude you from participating in the study:

- Exercising regularly: > 1-2 days/week depending on activity
- Dieting or intending to diet
- History of diabetes, heart disease or hypertension (high blood pressure) and/or taking medications for such conditions
- Pregnant or intending to become pregnant

The amount of radiation used in the study may expose an unborn child to significant risks. Therefore, if you are pregnant or intending to become pregnant you will be excluded from this study. If you are a woman of child bearing potential but not intending to get pregnant, you must agree to use appropriate contraceptive measures for the duration of the study. Medically acceptable contraceptives include: (1) surgical sterilization, (2) approved hormonal contraceptives (such as birth control pills, Depo-Provera, or Lupron Depot), (3) barrier methods (such as a condom or diaphragm) used with a spermicide, or (4) an intrauterine device (IUD). If you participate and become pregnant during the study you must notify Dr. William E. Kraus, M.D. or study staff immediately so that your participation in the study can be stopped.

- Orthopedic limitations, musculoskeletal disease and/or injury

Due to the nature of the study, and the potential of performing aerobic and/or resistance training exercises, persons with known joint, muscle, or other orthopedic limitations that restrict physical activity may be excluded. Examples of these limitations include shoulder problems (rotator cuff, etc.), chronic back and neck injuries, chronic knee, ankle or hip discomfort, sciatica and permanently torn muscles. Any muscle or joint condition that limits the ability to perform resistance training exercises or the ability to walk briskly for at least thirty (30) to forty-five (45) minutes may be cause for exclusion. If existing problems seem tolerable you may be asked to obtain permission from your physician before being included in the study.

- Allergic to xylocaine (anesthetic or numbing medicine)

Xylocaine 2% is used to anesthetize or numb the muscle biopsy incision site during Visit 4, Part 1 described below. Therefore anyone allergic to xylocaine will be excluded from the study.

- Unwillingness to complete the four (4) month control period (described below)
- Unwillingness to be randomized into one of four exercise groups (described below)

**WHAT DOES THE STUDY INVOLVE?**

If you agree to be in the study, you will be asked to sign this consent form. Each participant will begin the study with a four (4) month control period, maintaining your current lifestyle by making no changes to your current diet and/or exercise habits. The 4-month control period will begin once you have completed the following visits:

- **Visit 1:** You must FAST (nothing but water) for 12-hours before this visit. Daily medications may be taken. You will view a slideshow about the study and will be asked to sign the consent form. Then a sample of blood (one teaspoon) will be drawn to measure blood lipids, glucose and insulin to ensure you are eligible for the study. This visit will take place at the Duke Center For Living and will last approximately two (2) hours.
- **Visit 2:** You will undergo an exercise performance evaluation which involves exercising on a treadmill to a maximum effort during which time you will have a mouthpiece in your mouth to determine oxygen use through breathing. Blood pressure will be measured at rest and during the exercise phase of this test. For safety purposes your heart will be monitored by electrocardiogram (ECG). The ECG will be used to make sure you do not have any existing heart conditions and/or abnormalities. This visit will take place at the Duke Center For Living and will last approximately one (1) hour.
- **Visit 3:** You must FAST (no food or drink) for 12-hours prior to this visit. Water and daily medications may be taken.
  - **Part 1:** A Computerized Tomography (CT) Scan will be performed to measure body fat and muscle amounts as well as density in the abdomen, thigh and liver by x-ray. The CT scan will be done at Duke University Medical Center (South) and takes approximately one (1) hour including travel time. A parking pass will be provided for this visit.
  - **Part 2:** Percent body fat will be determined via the *BOD POD* ® method**.** You will sit in a fiberglass chamber containing a large viewing window while your body volume is measured using the *BOD POD* ® air displacement method. Spandex clothing and swim cap are required and will be provided by the study staff. This test may not be comfortable for anyone who has felt claustrophobic before but is generally well tolerated. This test takes approximately twenty (20) minutes.
  - **Part 3:** You will be given an RT3 Accelerometer device (similar in shape and size as a pager) to wear on your waistband for seven (7) consecutive days except while sleeping and showering. This device will record all physical activity done over the seven-day period. You will be asked to keep a log during the 7 days to help track the time in which the device was put on and taken off.
- **Visit 4:** You must FAST (nothing but water) for 12-hours prior to this visit. Daily medications may be taken. Please avoid products such as aspirin, ibuprofen, naproxen and any other anti inflammatory up to five (5) days before this visit to reduce the risk of excess bleeding from the biopsy site. This visit will take place at the Duke Center For Living and will take approximately four (4) hours to complete.
  - **Part 1:** After local anesthesia (xylocaine, 2%) is injected, a small incision will be made in the left thigh. Four to six small pieces of muscle, weighing approximately 40-50 mg each, the size of a pea, will be surgically removed. The incision site will be closed using steri strips.
  - **Part 2:** Your carbohydrate metabolism and insulin sensitivity will be evaluated by an intravenous glucose tolerance test (IVGTT). Glucose and insulin will be administered through a catheter (thin tube placed into your vein), one in each arm. Several blood samples will be drawn over a three (3) hour period. The blood samples taken throughout the 3 hour period total approximately one hundred (100) milliliters or three and one-third (3 1/3) ounces.
  - **Part 3:** During this visit you will complete five (5) questionnaires focusing on health, well-being, physical activity and quality of life. The questionnaires take approximately thirty (30) minutes to complete.
  - **Part 4:** During this visit the study nutritionist will spend about fifteen (15) minutes with you regarding your diet. You will be asked to recall your dietary habits. If the nutritionist is unable to meet with you, you will be contacted by phone and/or mail to complete this part of the visit.
  - **Part 5:** At the end of this visit you will have your body composition assessed by skinfold caliper and circumference measurements. Skinfold measurement will be taken at the triceps, abdomen, waist and thigh. Circumference measurements will be taken at the waist and hip. This part will take approximately ten (10) minutes.
- **Visit 5:** A maximal Isokinetic strength test will be done under the supervision of trained study staff at the Duke University Sports Medicine Michael W. Krzyzewski Human Performance Lab (K-Lab). This test determines maximal leg strength and fatigue. This test takes approximately thirty (30) minutes including driving time.

Please note that the visits described above may or may not occur in order. Please note that all visits take place during regular business hours, eight (8) AM to five (5) PM, and may require that you take time off from work. After completing Visits 1-5, the four (4) month control period will begin. At the end of the 4-month period you will return for end of control testing, Visits 2-5, once again, not necessarily in the order described. After completing the end of control testing participants will be randomized (like drawing a number out of a hat) into one (1) of four (4) exercise groups. The groups are:

- **Group 1:** Aerobic High Intensity/High Amount
  - Expending two-thousand (2000) kilocalories per week at a vigorous exercise intensity
- **Group 2:** Aerobic High Intensity/Moderate Amount
  - Expending approximately twelve-hundred (1200) kilocalories per week at a vigorous intensity
- **Group 3:** Resistance
  - Three (3) sets of eight (8) resistance training exercises performed on three (3) days each week
- **Group 4:** Aerobic High Intensity/Moderate Amount and Resistance
  - Combination of group 2 and group 3

Each group will exercise for a total of eight (8) months at the Duke Center For Living. The first two (2) – three (3) months will focus on slowly increasing the amount of exercise performed up to the individualized exercise prescription calculated. A sub-maximal test (using the head set and mouthpiece device used in Visit 2) will be done to determine a target heart rate range for participants randomized into a group that requires aerobic exercise Groups 1,2 and 4). All aerobic exercise will be done on either a treadmill or elliptical trainer and rarely a recumbent bicycle. During the 8-months, all aerobic exercise will be monitored and verified with a *Polar®* heart rate monitor and all resistance exercise will be monitored and verified by *Fitlinxx®* (a computerized resistance training partner). Please note that we ask all participants to exercise during staff supervised times at least half of the prescribed exercise sessions each week. Supervised coverage times occur Monday through Thursday from 6:30 – 8:00am and from 4:30 – 6:00pm. These times are subject to change.

At the end of the 8-month exercise-training period participants will complete Visits 2-5 for the third time. These tests will be followed by a two (2) week detraining period. During this 2-weeks participants will not exercise. Once the 2-weeks is over Visits 2-5 will be repeated for the fourth and last time except Visit 3, Part 1 (CT scan).

**HOW LONG WILL I BE IN THE STUDY?**

The study timeline is one (1) year or twelve (12) months (4-month control period plus the 8-month exercise training period). Participants can choose to stop participating at any time. However, if you decide to stop participating in the study, we will encourage you to talk to the study staff and/or doctor first.

Upon completion of the 8-month exercise study participants may be given the option to participate in a post-exercise training study. We will recruit up to fifty (50) men and women volunteers for this study. The follow-up study is completely voluntary. Participating will require participants to continue to wear a *Polar®* heart rate monitor for all aerobic exercise and will continue to use *Fitlinxx®* during all resistance exercise sessions for up to five (5) years. Participants will be asked to complete Visits 2-5 at six (6) months and on one (1) year and then yearly for up to 5 years of which Visit 4, Part 1 (muscle biopsy) will be optional.

In addition, participants may volunteer to be in a follow-up study. Volunteers will be contacted via phone to gain information regarding exercise habits and how things may have changed since participating in the original study as well as how one’s body may have changed over that time period. The purpose of the follow-up study is to evaluate the exercise habits of individuals after the eight-month structured and supervised exercise program. Information gained from this study will be used to evaluate why some individuals continue to exercise on their own, while others stop exercising. This information will be used to heighten the understanding and importance of a structured exercise program.

**WHAT ARE THE RISKS OF THE STUDY?**

There are certain risks associated with participating in the study. You may discuss these risks with the study doctor or your health care provider if you choose. The risks are:

- Participation in an exercise program may result in muscle, bone and/or joint soreness, discomfort and/or injury.
- Muscle biopsies from the thigh may result in momentary pain and discomfort, burning or bleeding, numbness, and rarely fainting or infection. Please notify Dr. William E. Kraus, M.D. or study staff if you have taken any product containing aspirin within five (5) days of the muscle biopsy as aspirin can increase bleeding risk. The incision site may leave a scar and muscle soreness may be present up to ten (10) days after the biopsy.
- Momentary discomfort, bleeding, bruising and rarely fainting or infection may result from the placement of two (2) catheters, one in each arm, one for the purpose of drawing blood samples and one for injecting glucose and insulin during the Intravenous Glucose Tolerance Test (IVGTT).
- If you take part in this research, you will have one or more medical imaging studies, which use radiation. The tests you will have include three single slice abdominal Computed Tomography (CT) scans, three single slice liver and gallbladder CT scans and three single slice thigh CT scans. To give you an idea about how much radiation you will get, we will make a comparison with an every-day situation. Everyone receives a small amount of unavoidable radiation each year. Some of this radiation comes from space and some from naturally occurring radioactive forms of water and minerals. This research (all three scans combined) gives your body the equivalent of about 4 extra months' worth of this natural radiation. The radiation dose we have discussed is what you will receive from this study only and does not include any exposure you may have received or will receive from other medical tests. The amount of radiation exposure during each of the Computerized Tomography (CT) scans may cause significant risks to an unborn child. Therefore, women of child bearing potential will have a blood pregnancy test using one (1) teaspoon of blood drawn by needle-stick, will be done within seventy-two (72) hours of each Computerized Tomography (CT) scan, and must be negative in order to continue participation. There may be other risks or side effects that are unknown at this time.

**ARE THERE BENEFITS TO TAKING PART IN THE STUDY?**

This study may not result in any benefit for participants; however, exercise training usually improves exercise performance and may improve risk factors for developing heart and blood vessel disease. Participation will provide new information on the optimum exercise program for improving risk factors, and thus may be beneficial to the general population.

Participants will receive one hundred ($100.00) dollars at completion of each of the four (4) muscle biopsy and intravenous glucose tolerance tests (Visit 3).

Participants will also receive three (3) months of free membership to the Duke Center For Living fitness center once the 8-month exercise training is completed, the *Polar®* heart rate monitor is returned and all exit testing has been done. You also have the opportunity to earn up to eight (8) more free months, one (1) free month of membership for each month that you are > 90% compliant during the eight (8) month exercise training phase.

**WILL MY INFORMATION BE KEPT PRIVATE?**

Study records that identify participants will be kept confidential as required by law. Federal Privacy Regulations provide safeguards for privacy, security, and authorized access. Except when required by law, participants will not be identified by name, social security number, address, telephone number, or any other direct personal identifier in study records disclosed outside of Duke University Health System (DUHS). For records disclosed outside of DUHS, you will be assigned a unique code number. The key to the code will be kept in a locked file in Dr. William E. Kraus’s office. As part of the study, Dr. Kraus and his study staff will report the results of study-related tests. While the information and data resulting from the study may be presented at scientific meetings or published in a scientific journal, identity will not be revealed.

Participant records may be reviewed in order to meet federal or state regulations. Reviewers may include representatives of the National Institutes of Health and the Duke University Health System Institutional Review Board. If any of these groups review research records, they may also need to review one’s entire medical record. Any significant findings developed during the course of this research, which may bear upon your condition or willingness to continue participation in the study, will be provided to you and your primary physician. The study results will be retained in your research record forever. The sponsor, National Institutes of Health, may further disclose the information from this study. If this information is disclosed to outside reviewers for audit purposes, it may be further disclosed by them and may not be covered by the federal privacy regulations.

**WHAT IF I HAVE A RESEARCH RELATED INJURY?**

Immediate necessary medical care is available at Duke University Medical Center in the event that you are injured as a result of your participation in this research study. However, there is no commitment by Duke University, Duke University Health System, Inc., or your Duke physicians to provide monetary compensation or free medical care to you in the event of a study-related injury.

For questions about the study or research-related injury, contact Dr. William E. Kraus at (919) 660-6613 during regular business hours and at (919) 970-7682 after hours and on weekends and holidays.

**WHAT ARE THE COSTS?**

There will be no costs to the participant as a result of being in this study. However, routine medical care (care that would be received whether in the study or not) will be charged to the participant or the corresponding insurance company. If necessary, the insurance company may be contacted to discuss this further.

**WHAT ABOUT MY RIGHTS TO DECLINE PARTICIPATION OR WITHDRAW FROM THE STUDY?**

You may choose not to be in the study, or, if you agree to be in the study, you may withdraw from the study at any time. If you withdraw from the study, no new data will be collected for study purposes other than data needed to keep track of your withdrawal. If you withdraw from the study all data and samples that have already been collected for study purposes will be used unless a written request for all data to be destroyed is sent to Dr. William E. Kraus or his study staff. All requests should be mailed to William E. Kraus at DUMC Box 3022, Durham, NC 27710. Participants who withdraw from the study will discontinue all study procedures and will not receive compensation for the uncompleted muscle biopsy/intravenous glucose tolerance test nor the free 3-month fitness center membership.

**WHOM DO I CALL IF I HAVE QUESTIONS OR PROBLEMS?**

For questions about the study or a research-related injury, contact Dr. William E. Kraus at (919) 660-6613 or the Duke University Health System Institutional Review Board (IRB) Office at (919) 668-5111.

**STATEMENT OF CONSENT**

"The purpose of this study, procedures to be followed as well as risks and benefits have been explained to me. I have been allowed to ask questions, and my questions have been answered to my satisfaction. I have been told whom to contact if I have additional questions. I have read this consent form and agree to be in this study, with the understanding that I may withdraw at any time. I have been told that I will be given a signed copy of this consent form."

__________________________________________ ____________________

Signature of Subject Date

__________________________________________ ____________________

Signature of Person Obtaining Consent Date
